# Outcomes of vedolizumab therapy in patients with immune checkpoint inhibitor–induced colitis: a multi-center study

**DOI:** 10.1186/s40425-018-0461-4

**Published:** 2018-12-05

**Authors:** Hamzah Abu-Sbeih, Faisal S. Ali, Dana Alsaadi, Joseph Jennings, Wenyi Luo, Zimu Gong, David M. Richards, Aline Charabaty, Yinghong Wang

**Affiliations:** 1 Department of Gastroenterology, Hepatology and Nutrition, The University of Texas MD Anderson Cancer Center, 1515 Holcombe Blvd, Houston, TX 77030 USA; 2 Division of Gastroenterology, MedStar-Georgetown University Hospital, Washington, DC, USA; 3 Department of Pathology/Laboratory Medicine, The University of Texas MD Anderson Cancer Center, Houston, TX USA; 4 Department of Hematopathology, The University of Texas MD Anderson Cancer Center, Houston, TX USA

**Keywords:** Vedolizumab, Colitis, Diarrhea, Immunotherapy, Immune checkpoint inhibitor

## Abstract

**Background:**

Immune-mediated diarrhea and colitis (IMDC) can limit immune checkpoint inhibitors (ICIs) treatment, which is efficacious for advanced malignancies. Steroids and infliximab are commonly used to treat it. These agents induce systemic immunosuppression, with its associated morbidity. We assessed clinical outcomes of vedolizumab as an alternative treatment for IMDC.

**Methods:**

We analyzed a retrospective case series of adults who had IMDC refractory to steroids and/or infliximab and received vedolizumab from 12/2016 through 04/2018.

**Results:**

Twenty-eight patients were included. The median time from ICI therapy to IMDC onset was 10 weeks. Fifteen patients (54%) had grade 2 and 13 (46%) had grade 3 or 4 IMDC. Mucosal ulceration was present in 8 patients (29%), and nonulcerative inflammation was present in 13 (46%). All patients had features of active histologic inflammation; 14 (50%) had features of chronicity, and 10 (36%) had features of microscopic colitis concurrently. The mean duration of steroid therapy was 96 days (standard deviation 74 days). Nine patients received infliximab in addition to steroids and their IMDC was refractory to it. Among these, the duration of steroid use was 131 days compared with 85 days in patients who did not receive infliximab. Likewise, patients who failed infliximab before vedolizumab had a clinical success rate of 67% compared to 95% for patients that did not receive infliximab. The median number of vedolizumab infusions was 3 (interquartile range 1–4). The mean duration of follow-up was 15 months. Twenty-four patients (86%) achieved and sustained clinical remission. Repeat endoscopic evaluation was performed in 17 patients. Endoscopic remission was attained in 7 (54%) of the 13 patients who had abnormal endoscopic findings initially; 5/17 patients (29%) reached histologic remission as well.

**Conclusions:**

Vedolizumab can be appropriate for the treatment of steroid-refractory IMDC, with favorable outcomes and a good safety profile.

**Electronic supplementary material:**

The online version of this article (10.1186/s40425-018-0461-4) contains supplementary material, which is available to authorized users.

## Background

Cancer therapy has witnessed a paradigm shift with the advent of immune checkpoint inhibitors (ICIs), particularly cytotoxic T-lymphocyte activator-4 (CTLA-4), programmed cell death protein 1 (PD-1), and programmed cell death ligand 1 (PD-L1) inhibitors [1–4]. By inhibiting checkpoints that are involved in regulating T-cell activation, ICIs have allowed augmentation of immunologic response against tumor cells, which in turn has improved survival outcomes, particularly in patients with small cell lung carcinoma, renal cell carcinoma, and melanoma [3, 5–8]. Multiple clinical trials are underway to assess the efficacy of ICIs in various malignancies, and the list of malignancies that respond to ICIs is expected to grow.

However, although ICIs are a promising cancer therapy, they also come with challenges. ICIs cause a widespread activation of T-cells that is not tumor-specific [9]. This activation, coupled with the depletion of regulatory T-cells, which is thought to occur with CTLA-4 therapy, causes T-cells to attack various organ systems, leading to a spectrum of adverse events commonly known as immune-related adverse events (irAEs). IrAEs can be very mild, requiring only observation and symptomatic care, or they can be life-threatening, in which case inadequate management can prove fatal [10, 11]. Although irAEs can theoretically affect any organ system, they have most commonly been found to affect the gastrointestinal (GI) tract, endocrine system, lungs, liver, skin, and, rarely, the eyes and peripheral nervous system. GI-irAEs are the most commonly reported grade 3–4 irAEs [10]. GI-irAEs range from mild diarrhea to severe, life-threatening enterocolitis, which can be challenging to treat [12].

Mild (grade 1) GI-irAEs are managed symptomatically with a watchful waiting strategy when the adverse event is thought to be self-limiting. GI-irAEs of moderate severity (grade 2–3 diarrhea/enterocolitis) prompt medical management. Corticosteroids are generally used as the first-line therapy and have recently been recommended in a consensus practice guideline statement [10, 13]. GI-irAEs that are refractory to high-dose corticosteroids are managed with alternative or add-on immunosuppressive therapy. Infliximab is the most validated second-line treatment [10, 14]. However, immunosuppressive therapy may adversely affect the antitumor efficacy of ICI therapy [15]. Furthermore, systemic immunosuppressive therapy is associated with various debilitating adverse events, including infections, diabetes mellitus, osteoporosis, myopathy, adrenal insufficiency, mood disorders, and cataracts [16, 17]. Moreover, a subset of patients suffer from GI-irAEs that are refractory to second-line immunosuppressive therapy.

Thus, there is a need for an alternative effective treatment for GI-irAE that does not hamper the antitumor effect of ICIs. Vedolizumab is a gut-targeted α4β7 integrin antibody approved for the treatment of inflammatory bowel disease (IBD), which is a disease that shares a considerable number of clinical and molecular features with ICI-induced colitis [18, 19]. Also, it is a humanized monoclonal IgG1 antibody that blocks the interaction between α_4_β_7_ integrin found on the surface of T-cells and mucosal vascular addressin cell adhesion molecule 1 **(**MAdCAM-1) expressed on endothelial surface of venules within the GI tract and associated lymphoid tissue, which prevents leukocyte binding to the endothelial surface and its extravasation into affected tissue, enabling selective GI immunosuppression [20, 21]. We report our experience with vedolizumab in treating GI-irAEs that failed to resolve with first- and second-line immunosuppressive therapy.

## Methods

### Study design and population

This retrospective, descriptive, multi-center study was conducted after approval was obtained from the Institutional Review Boards at The University of Texas MD Anderson Cancer Center and Medstar-Georgetown University Center. Patients included in our study (1) developed ICI-induced colitis that was resistant to steroids, (2) received vedolizumab between December 2016 and April 2018 owing to histopathologic evidence of ICI-induced enterocolitis, and (3) had clinical or endoscopic follow-up after vedolizumab therapy. Pertinent data were extracted from electronic patient health records and endoscopy databases. In addition to patient demographics, we extracted information relating to the endoscopic procedures performed as a part of the colitis workup (with or without biopsy), as well as pathologic findings of biopsies. Additional data regarding serum and fecal inflammatory markers, ICI therapy dose and duration, and duration of treatment for the GI-irAE were retrieved. No patient consent was required owing to the retrospective nature of the study and no risk posed to patients.

### Clinical data and treatment for colitis

Information about each patient’s cancer type, stage, type of ICI therapy, and number of ICI infusions was extracted. The date of GI-irAE onset was considered the initial documentation of diarrhea. Diarrhea was graded in accordance with the Common Terminology Criteria for Adverse Events (CTCAE version 5.0; Additional file 1: Table S1) [22]. Regarding treatment for the GI-irAE, date of corticosteroid initiation was recorded, along with the date on which a pause in steroid therapy occurred. The cumulative duration of steroid therapy was reported, excluding the break between different treatment courses (e.g., a pause during which a diarrhea flare-up occurred). The GI-irAE was considered steroid-refractory when (1) symptoms only partially improved after the patient received the highest-dose corticosteroid therapy (2 mg/kg prednisone or equivalent), (2) symptoms relapsed upon tapering or discontinuing steroids, or (3) steroid dependency signs and symptoms occurred upon tapering of corticosteroids. Immunosuppressive medications that were added on to the steroid regimen were also recorded. We calculated the duration from first vedolizumab infusion to improvement of clinical symptoms to grade 1 or below as one of the outcomes. Clinical remission of symptoms was defined as sustained resolution of diarrhea to grade 1 or lower after vedolizumab therapy.

### Endoscopic analysis

The type, timing, frequency, and findings of endoscopic procedures were recorded. Endoscopic findings were reported according to the appearance of the observed mucosa and were classified as (1) ulceration, (2) nonulcerative inflammation (erythema, edema, decreased/altered vascular pattern, friability, and/or erosion), or (3) normal appearance. The location of inflammatory findings as well as the pattern of inflammation observed was recorded as one of the following three categories: (1) extensive inflammation (involving the right colon), (2) localized inflammation (limited to the left colon), or (3) isolated small bowel inflammation. If the GI tract was biopsied during diagnostic endoscopy, information regarding biopsy findings was extracted as well. Clinical remission was defined as a return to baseline bowel movement pattern when steroids were discontinued, with a sustained remission for the follow-up duration of our study. Endoscopic remission was defined as Mayo endoscopic subscore of 0 or 1 after vedolizumab therapy [23].

### Histologic characterization

Histopathologic slides of biopsied tissue from the terminal ileum, duodenum, and colon were examined by two board-certified pathologists, who confirmed the diagnosis of colitis. The severity of chronic or active inflammation was separately graded. Additionally, the severity of inflammation before and after interventions was compared for each individual patient.

Active inflammation was characterized by neutrophilic inflammation such as increased neutrophils in the lamina propria, cryptitis, and cryptic abscess with or without erosion or ulceration. A lymphocytic colitis pattern was defined as increased intraepithelial lymphocytes (> 20 per 100 enterocytes). A collagenous colitis pattern was defined as thickened basement membrane with increased lymphocytes and plasma cells in the lamina propria (intraepithelial lymphocytes may or may not reach the level of a lymphocytic colitis pattern) in well-oriented biopsy fragments.

Signs of chronicity included basal lymphoplasmacytosis, markedly altered architecture, or Paneth cell metaplasia. Infectious etiologies such as fungi, acid-fast bacilli, or cytomegalovirus were ruled out by histologic analysis, special stains, or immunohistochemistry whenever necessary. Histologic response was defined as improvement of active inflammation features at the last follow-up biopsy after vedolizumab therapy.

### Inflammatory markers

Evaluated inflammatory markers included fecal lactoferrin, fecal calprotectin, and C-reactive protein (CRP). Fecal calprotectin levels at the onset of symptoms, as well as before and after vedolizumab therapy, were recorded. In addition, CRP and fecal lactoferrin levels before and after vedolizumab therapy were recorded. We employed descriptive statistics for reporting of our findings.

### Vedolizumab therapy

The decision to proceed with additional immunosuppressive therapy besides corticosteroids was based on the clinical judgment of the primary gastroenterologist and a consensus with the primary oncology team. The timing and frequency of diagnostic endoscopy procedures was recorded. The timing, dose, and frequency of vedolizumab infusions were recorded. In patients with non-diagnostic symptoms or a complex differential diagnoses, endoscopic and histologic findings were used as evidence to guide GI-irAE therapy.

## Results

### Baseline characteristics

A total of 28 patients were enrolled. Twenty-five patients were from MD Anderson and three were from Medstar-Georgetown Medical Center. Twenty patients (71%) were male and 25 (89%) were of white race. The mean age at initiation of ICI therapy was 63 years (standard deviation [SD] 10 years). Patient characteristics and cancer- and treatment-related data are summarized in Table 1. None of the patients had a history of IBD. All patients had been treated with chemotherapy or targeted therapy before the decision to initiate ICI therapy was made. PD-1/PD-L1 inhibitors were the most commonly used agents. The median number of ICI infusions was 3 (interquartile range [IQR] 1–36). Melanoma (7 patients, 25%) was the most common malignancy. Twenty-two patients (79%) suffered from a stage IV malignancy. The median time from the first ICI infusion to onset of diarrhea was 10 weeks (IQR 1–70). All patients had diarrhea as the presenting symptom; 15 had grade 2 and 13 had grade 3 or 4. Fourteen patients (50%) suffered from abdominal pain, and 11 (39%) had blood or mucous in the stool. ICI therapy was discontinued in all patients before the initiation of corticosteroid therapy. Seven patients developed one or more non-GI irAEs; skin rash (*n* = 4), thyroiditis (*n* = 1), adrenalitis (*n* = 1), pneumonitis (*n* = 1), myositis and arthralgia (*n* = 2), lipase elevation (*n* = 1). Only two patients had concurrent colitis and non-GI adverse events; one had diabetes type I and lipase elevation and one had arthralgia. In both of them, no impact from vedolizumab was observed on non-GI adverse events. The non-GI adverse events in the other 5 patients resolved before the administration of vedolizumab.

**Table 1:** Patient clinical characteristics (*n* = 28)

Characteristic	No. of patients (%)
Mean age, years (SD)	63 (10)
Male sex	20 (71)
Cancer type	
Melanoma	7 (25)
Renal cell carcinoma	4 (14)
Prostate carcinoma	4 (14)
Urothelial	3 (11)
Other solid tumors	10 (36)
Cancer stage	
III	6 (21)
IV	22 (79)
ICI type	
CTLA-4	8 (29)
PD-1/L1	12 (43)
Combination	8 (29)
Median no. of ICI infusions (IQR)	3 (1–36)
Median time to diarrhea onset, weeks (IQR)	10 (1–70)
Peak CTCAE grade of diarrhea	
2	15 (54)
3–4	13 (46)
Peak CTCAE grade of colitis	
1–2	16 (57)
3–4	12 (43)
Colitis symptoms	
Abdominal pain	14 (50)
Blood or mucous in stool	11 (39)
Other adverse events	
Dermatological	4 (14)
Endocrine	2 (7)
Musculoskeletal	2 (7)
Pulmonary	1 (4)
Pancreatic	1 (4)

Abbreviations: *SD*, standard deviation; *ICI*, immune checkpoint inhibitor; *IQR*, interquartile range; *CTCAE*, Common Terminology Criteria of Adverse Events

### Diagnostic workup

#### Endoscopic evaluation

**Fig. 1:**
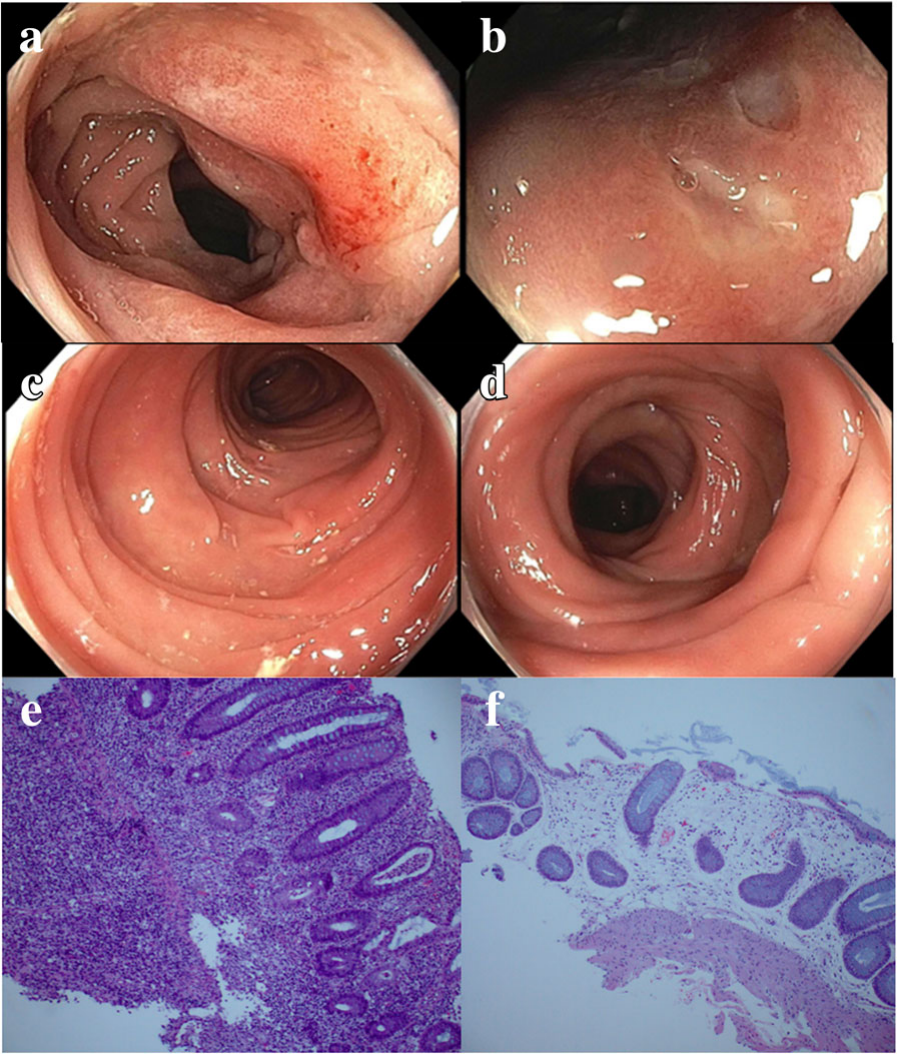
Representative endoscopic and histologic findings before and after vedolizumab therapy in patients who had good response. (**a**, **b**) Endoscopic presentations before vedolizumab therapy showing diffuse erythema and focal punctate mucosal ulcerations. (**c**, **d**) Endoscopic presentations from the same patients after vedolizumab therapy showing normal mucosa. (**e**) Before vedolizumab therapy, chronic active colitis with cryptitis and crypt abscesses in the background of lamina propria expansion by lymphoplasmacytic infiltration, basal lymphoplasmacytosis, and architecture distortion. (**f**) After vedolizumab therapy, chronic colitis with architecture distortion and glandular dropout without active colitis. Chronic infiltrates in the lamina propria were markedly reduced to almost normal

**Fig. 2:**
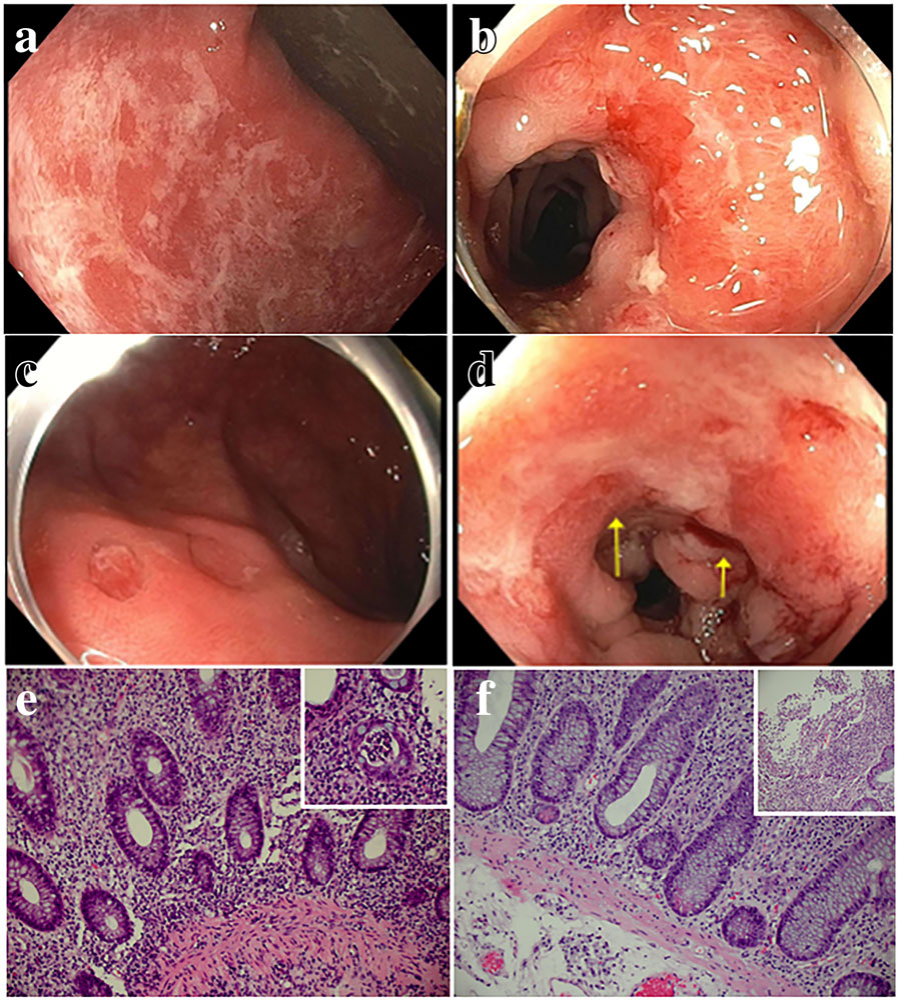
Representative endoscopic and histologic findings before and after vedolizumab treatment in patients who failed treatment. (**a**, **b**) Endoscopic presentation before vedolizumab therapy showing diffuse erythema, edema and mucosal ulcerations. (**c**, **d**) Endoscopic presentation from the same patients after vedolizumab therapy showing persistence of mucosal ulceration and erythema. (**e**) Before vedolizumab therapy, chronic active colitis with basal plasmacytosis and expansion of lamina propria by lymphohistiocytes. In the top right corner, there are focal areas of neutrophil infiltration in the crypt lumen forming cryptitis and crypt abscess. (**f**) After vedolizumab therapy, chronic active colitis with basal plasmacytosis and Paneth cell metaplasia. In the top right corner, there are focal areas of active colitis with focal ulceration and granulation tissue

All patients underwent diagnostic endoscopic evaluation with biopsy. Table 2 summarizes findings of pertinent diagnostic studies. Endoscopic findings before and after vedolizumab therapy are demonstrated in Figs. 1 and 2. Most patients (22, 79%) underwent diagnostic colonoscopy, although six patients underwent flexible sigmoidoscopy for the initial endoscopic evaluation, and two underwent an upper endoscopy in addition to a colonoscopy. Regarding endoscopic evidence of inflammation, 13 patients (46%) had nonulcerative inflammation, 8 (29%) had mucosal ulceration, and 7 (25%) had normal endoscopic features. The distribution of endoscopic inflammation was extensive (involving both the right and left colon) in 14 patients (50%), whereas 5 (18%) had inflammation involving only the left colon, and 2 (7%) had isolated small bowel involvement. In terms of follow-up endoscopic examination after vedolizumab therapy, 11 patients had none and 17 patients underwent one or more follow-up endoscopies. The median number of endoscopic procedures was 2 (IQR 1–7).

**Table 2:** Patient diagnostic evaluation data (*n* = 28)

Characteristic	No. of patients (%)
Endoscopy and histologic features	
Type of endoscopy	
Colonoscopy	22 (79)
Flexible sigmoidoscopy	6 (21)
Endoscopic findings on initial evaluation	
Ulceration	8 (29)
Nonulcerative inflammation	13 (46)
Normal	7 (25)
Endoscopic distribution	
Extensive	14 (50)
Left colon only	5 (18)
Isolated small bowel	2 (7)
Histological inflammation on initial evaluation	
Active features	28 (100)
Chronic features	14 (50)
Microscopic	10 (36)
Median no. of endoscopic procedures (IQR)	2 (1–7)
Diagnostic laboratory studies	
Mean duration of laboratory follow-up, months (SD)	3 (4)
Positive fecal lactoferrin at onset of diarrhea^a^	23 (100)
Positive fecal lactoferrin after vedolizumab therapy^b^	11 (79)
Mean fecal calprotectin value at onset of diarrhea μg/g (SD)^c^	329 (276)
Mean fecal calprotectin value at follow-up μg/g (SD)^d^	218 (262)

Abbreviations: *IQR*, interquartile range; *SD*, standard deviation.

^a^Lactoferrin was initially measured for 23 patients

^b^Lactoferrin was measured at follow-up for 14 patients

^c^Calprotectin was initially measured for 19 patients

^d^Calprotectin was measured at follow-up for 13 patients

#### Histologic evaluation

All patients underwent biopsies of the colon, terminal ileum, and duodenum (when EGD evaluation was done), irrespective of endoscopic evidence of inflammation. Histopathologic presentation before and after vedolizumab therapy is shown in Figs. 1 and 2. Upon initial histologic evaluation, 28 patients (100%) had features of active inflammation and 14 (50%) had features of chronic inflammation. Two major patterns of inflammation were observed, one with active colitis superimposed on chronic colitis, and a second pattern characterized by inflammation in the spectrum of lymphocytic colitis and collagenous colitis. Fourteen patients had features of chronicity such as architecture distortion, basal lymphoplasmacytosis, and Paneth cell metaplasia. Granulomatous inflammation was not observed. Ten patients had a microscopic inflammation pattern in the spectrum of lymphocytic colitis (eight patients) or collagenous colitis (two patients). Biopsies from eight patients also showed terminal ileitis evidenced by focal villitis and architectural distortion. Markedly increased mitotic activity and apoptosis of epithelial cells were observed in nine patients.

#### Laboratory studies

**Fig. 3:**
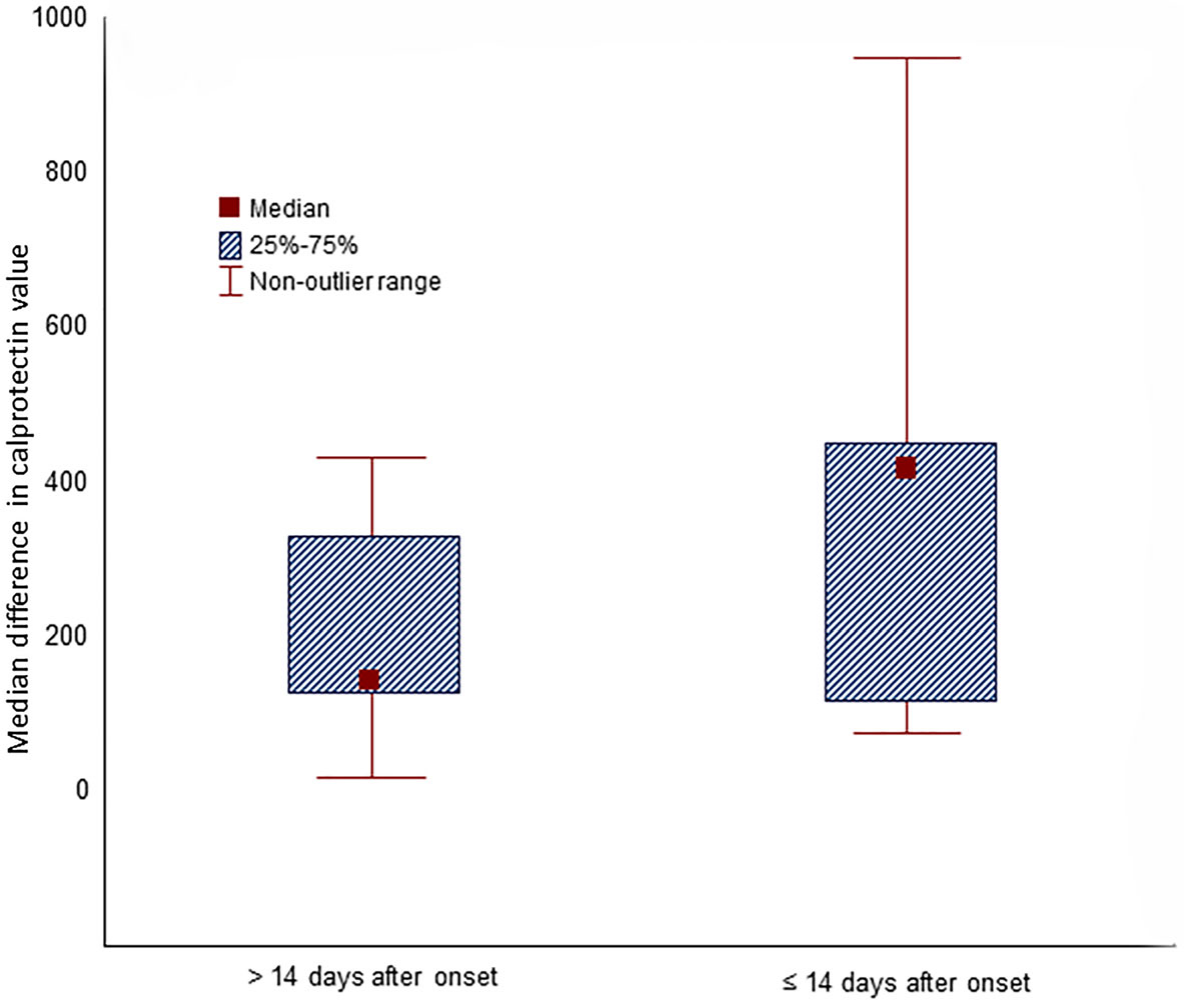
Decrease in calprotectin values after vedolizumab/infliximab therapy according to time from onset to treatment initiation

Infectious workup was done for all patients to rule out an infectious cause of diarrhea, including *Clostridium difficile*. Only one patient had pathologic evidence of cytomegalovirus inclusions, which was regarded as a super infection and treated with ganciclovir. The median CRP level at the time of symptom onset was 19.4 (IQR 4.59–131.95). After vedolizumab therapy initiation, the median CRP level was 10.87 (IQR 1.45–168.73). The mean fecal calprotectin value at the time of onset was 329 μg/g (SD 276 μg/mg), whereas the mean fecal calprotectin at follow-up was 218 μg/g (SD 262 μg/mg). Additionally, fecal lactoferrin assay results at symptom onset were positive in all 23 patients who were tested. A follow-up fecal lactoferrin assay was performed in 14 patients, and it was positive in 11 (79%). The mean follow-up duration for laboratory tests was 3 months. Figure 3 demonstrates the difference in calprotectin values before and after vedolizumab/infliximab therapy, stratified by the mean duration from symptom onset to vedolizumab/infliximab initiation.

### Treatment for colitis and outcomes

#### Treatment for ICI-induced colitis

The initial therapeutic management for colitis in all patients who received corticosteroid therapy was intravenous methylprednisolone, followed by oral prednisone. Four patients were treated with steroid enemas in addition to corticosteroid therapy. The mean duration of corticosteroid therapy was 96 days (SD 74; Table 3). All patients were considered to have a steroid-refractory GI-irAE. In all patients, at least one corticosteroid tapering trial failed, with a maximum of three attempts. Corticosteroids were reinitiated after a failed attempt at tapering, either intravenously in cases that were severe enough to prompt an emergency room visit or as a step-up of the oral dose.

**Table 3:** Treatment for colitis and outcomes (*n* = 28)

Characteristic	No. of patients (%)
Treatment	
Mean overall duration of steroid therapy, days (SD)	96 (74)
Diarrhea refractory to steroids	28 (100)
Infliximab therapy	9 (32)
Median no. of infliximab doses (IQR)	2 (1–3)
Recurrent symptoms while receiving infliximab	9 (100)
Median no. of vedolizumab doses (IQR)	3 (1–4)
Outcomes	
Median duration from 1st vedolizumab to improvement, days (IQR)	5 (1–30)
Mean duration of endoscopic follow-up, months (SD)	6 (4)
Clinical remission at last follow-up	24 (86)
Endoscopic remission at last follow-up^a^	7 (54)
Histologic remission at last follow-up^a^	5 (29)

Abbreviations: SD, standard deviation; IQR, interquartile range.

^a^Repeat endoscopic and histologic evaluations were performed in 17 patients. However, endoscopic remission was counted for only 13 patients who had abnormal endoscopic findings initially and had repeat evaluation

Nine patients in our cohort received a trial of mesalamine before corticosteroid therapy, with unsatisfactory response. The decision to administer infliximab was based on the clinical judgment of the consulting gastroenterologist. Nine of the 28 patients received infliximab in addition to corticosteroid therapy (Table 3). Five of these nine patients had also undergone an unsuccessful trial of mesalamine in addition to corticosteroid therapy. The median number of infliximab infusions was two (IQR 1–3). Infliximab therapy was deemed to have failed in all patients owing to persistent or recurrent symptoms after 1 month of infliximab therapy.

Vedolizumab was given following the same standard as when used to treat IBD, at 300 mg for each infusion via the standardized IBD infusion schedule. Patients received a median of three doses of vedolizumab (IQR 1–4). One patient developed a skin rash after vedolizumab infusion, which was thought to be caused by drug allergy, and that patient was switched to infliximab therapy. Additionally, one patient developed diffuse joint pain after one dose of vedolizumab, which led to discontinuation of vedolizumab, but the patient achieved clinical remission of GI irAE.

#### Follow-up and disease outcome

The mean duration of follow-up was 15 months. The median duration from first vedolizumab infusion to improvement of symptoms was 5 days (IQR, 1–30). The mean duration of endoscopic follow-up was 6 months (SD 4). Twenty four of the 28 patients (84%) had sustained clinical remission. Seven of the 13 patients (54%) who had abnormal endoscopic findings initially had achieved endoscopic remission on repeat exam. Regarding histologic inflammation before and after treatment of colitis, 17 patients had biopsy results available after vedolizumab therapy. Decreased acute and chronic inflammation was found on the biopsy specimens of five patients, whereas 12 patient biopsy specimens showed no significant difference compared with the pretreatment biopsy specimens. Two patients whose specimens were in the spectrum of microscopic colitis had biopsy results available after treatment, and these did not show significant improvement. Vedolizumab therapy failed clinically in four patients. One patient with lymphocytic colitis had achieved clinical and histologic remission after four doses of infliximab and three doses of vedolizumab, but this patient developed recurrent diarrhea after 3 months, with histologically confirmed recurrent lymphocytic colitis.

Nonulcerative inflammation was the most common endoscopic finding among patients in clinical remission (Table 4). Furthermore, patients who achieved clinical remission had lower mean fecal calprotectin levels at the time of symptom onset (299 μg/g compared with 586 μg/g) and a shorter mean overall disease course compared with patients who did not achieve clinical remission (5 months compared with 8 months). The ICI agent used, number of vedolizumab doses, and grade of diarrhea did not differ between those who achieved clinical remission and those who did not. Repeat endoscopy was performed at 6-month follow-up in 13 patients, and patients who did not experience clinical remission with vedolizumab therapy had more mucosal ulceration than did patients who achieved clinical remission (75% compared with 8%). Patients who achieved clinical remission with vedolizumab had more dramatic improvement in active histologic inflammation (100 to 69%) than did those who did not (100 to 100%).

**Table 4:** Vedolizumab therapy outcomes and clinical characteristics (n = 28)

Characteristic	Clinical remission, No. (%)	Clinical failure, No. (%)
Total no. of patients	24	4
Checkpoint inhibitor type		
CTLA-4	6 (25)	2 (50)
PD-1/L1	11 (46)	1 (25)
Combination	7 (29)	1 (25)
Mean duration of steroid therapy, days(SD)	95 (79)	99 (52)
Median time from symptom onset to vedolizumab/infliximab therapy, days (IQR)	19 (6–152)	9 (4–67)
Median no. of vedolizumab doses (IQR)	3 (1–4)	3 (1–4)
Mean fecal calprotectin level at time of onset μg/g (SD)	299 (236)	586 (584)
Peak grade of diarrhea		
2	12 (50)	2 (50)
3–4	12 (50)	2 (50)
Initial endoscopic findings		
Ulceration	6 (25)	3 (75)
Nonulcerative inflammation	12 (50)	0 (0)
Normal	6 (25)	1 (25)
Initial histologic findings		
Active features	24 (100)	4 (100)
Chronic features	10 (42)	4 (100)
Microscopic	9 (38)	1 (25)
Mean overall duration of disease months (SD)	5 (3)	8 (5)
Mean fecal calprotectin level after vedolizumab therapy μg/g (SD)	187 (108)	270 (487)
Last repeat endoscopic findings^a^		
Ulceration	1 (8)	3 (75)
Nonulcerative inflammation	6 (46)	0 (0)
Normal	6 (46)	1 (25)
Active features on last repeat histologic analysis^a^	9 (69)	4 (100)

Abbreviations: *SD*, standard deviation; *IQR*, interquartile range

^a^Repeat endoscopy and histologic analysis was performed in 13 patients with clinical remission

Patients who failed infliximab therapy prior to vedolizumab received steroid treatment for 131 days (SD, 74) compared with those who did not receive infliximab (85 days; SD, 75) (Additional file 1: Table S2). Additionally, patients who failed infliximab therapy required a median of 3 infusions of vedolizumab to achieve satisfactory results compared with a median of 2 infusions in those who did not receive infliximab. Clinical remission was attained in 67% of patients who failed infliximab compared with 95% of patients who did not receive infliximab. Active histological features of inflammation were persistent in 67% of patients who failed infliximab in contrast to 32% in patients who received no infliximab therapy. The clinical remission of colitis was achieved in 90% of patients who had microscopic colitis compared with 83% of patients who had active and/or chronic histology features (Additional file 1: Table S3). No difference were observed in clinical features of patients who had microscopic colitis on histology and those who did not.

## Discussion

The management of irAEs has been constructed based on strategies that were not evidence-based. Only recently have standardized guidelines surfaced regarding the management of irAEs. The current study summarizes our initial experience with vedolizumab as an alternative therapy for GI-irAEs refractory to first- and second-line immunosuppressive therapy. This case series from two tertiary institutions adds to the emerging body of evidence on the utility of vedolizumab for GI-irAEs. In our cohort, vedolizumab led to clinical remission in 84% of patients with steroid-refractory ICI-induced colitis and endoscopic remission in 54% of patients. A previous case report and a case series also reported effectively treating GI-irAEs with vedolizumab [24, 25]. The case series consisted of patients who had not achieved symptomatic remission with steroid therapy, except for one patient, who had not responded to either corticosteroid therapy or infliximab therapy prior to initiation of vedolizumab. Nine patients (32%) in our cohort did not respond to infliximab or corticosteroids before receiving vedolizumab.

Gastroenterologists were commonly involved in the treatment of this dismal entity since the approval of ipilimumab in 2011. Moreover, they used similar treatment strategies as those used for IBD. As the treatment for IBD evolved, more treatment options have been used to treat ICI-enterocolitis from this field. In 2014, vedolizumab was approved for the treatment of IBD [18, 19]. It is a humanized monoclonal IgG1 antibody that targets an integrin subtype known as lymphocyte Peyer patch adhesion molecule (LPAM), which mediates homing of a subset of CD4-T cells to the GI tract, hence enabling selective GI immunosuppression [20, 21]. Additionally, vedolizumab is specific to the α4 and β7 subunit of LPAM and does not bind to other integrins that are involved in homing of lymphocytes to other organs.

Patients in our case series had developed GI-irAEs that were refractory to steroid therapy. Although some of our patients were experiencing partial response from previous treatment at the time of vedolizumab infusion, the rationale behind proceeding with further therapy was based on individual patient history of relapses during steroid tapering or discontinuation, or histologic evidence of ongoing disease activity. Long-term steroid use is undesirable owing to the degree of immunosuppression and risk of steroid-related side effects such as infections, diabetes, and cataracts etc. Although the initiation of treatment for the GI-irAE was in concordance with the norm and may have achieved partial response, it was not feasible to taper off corticosteroids completely in our patients, which prompted additional immunosuppressive therapy with infliximab in some patients. Patients included in our study did not achieve remission from infliximab and mesalamine, as evidenced by symptoms, endoscopic findings, and/or histologic findings, and these patients therefore switched to vedolizumab therapy as an alternative option. The remaining patients received vedolizumab alone, without a trial of infliximab or mesalamine after steroid failure. In our study, we did not observe any effect from vedolizumab on non-GI irAEs in the two patients that had concurrent GI and non-GI adverse events.

In patients suffering from an ICI-induced GI-irAE that is refractory to corticosteroid therapy, treatment with infliximab is recommended [10]. Although some patients respond adequately to this treatment strategy, there is paucity of data regarding the effects of infliximab therapy in combination with corticosteroids on the antitumor activity of ICIs. Also unknown is the impact of the combination of immunosuppression with steroids and infliximab on overall survival and the risks of these agents in immunocompromised patients with active cancer who begin potential long-term use of anti-TNFα therapy.

Severe irAEs are speculated to be a potential indicator of improved tumor response to ICI therapy, although studies have reported conflicting results [8, 26–28]. If this proves to be the case, then a GI-targeted immunosuppressant should be a safer and a more favorable option over other immunosuppressants to avoid hampering tumor response to ICI therapy. The safety profile of vedolizumab seems favorable to systemic immunosuppression with anti-TNFα agents given that its mechanism of action is limited to lymphocyte trafficking in the GI tract. Safety data from six clincial trials showed that vedolizumab is not associated with increased risks of serious infections or malignancy [29]. Finally, vedolizumab should be considered to treat ICI-induced colitis in patients with a contraindication for anti-TNFα agents, such as latent tuberculosis (which was the case in one of our patients) or hepatitis B. Although the comparative efficacies of vedolizumab and infliximab for ICI-induced colitis have not been studied, the current case series suggests that vedolizumab may be a more effective agent than infliximab for inducing remission in ICI-induced colitis, or at least a very effective alternative to anti-TNFα therapy as a first-line biologic after steroid failure and as a second-line biologic in anti-TNFα primary nonresponders. The gut-specific mechanism of action, the safety profile, the quick onset of action, and the efficacy of vedolizumab all make this drug an attractive agent for the treatment of GI-irAEs. Noteworthy, this treatment approach highlights the importance of coordination between oncologists and other subspecialties to treat irAEs effectively and to help in evolving more treatment options.

Compared with those in previously reported series [24, 25], our patients had higher initial diarrhea grades. This is in concordance with the fact that GI symptomatology does not correlate with the degree of gut inflammation [30], and hence using symptoms to quantify the degree of inflammation may not be the most accurate approach to grading irAEs. Currently, irAEs are graded according to CTCAE. The evidence to support the claim that CTCAE optimally stratifies irAEs on the basis of their severity and outcomes is lacking. Nonetheless, CTCAE remains the widely accepted modality for grading irAEs, until a better, more fitting system surfaces. This highlights the need for a standardized system that is tailored to the clinical and histopathologic findings of ICI-induced irAEs.

A subset of our patients did not have any obvious inflammatory findings on colonoscopy analysis but did have inflammatory changes according to histopathologic assessment of biopsied mucosa that appeared normal [31]. This highlights the importance of biopsies of the colon and the small bowel when assessing patients for ICI-induced enterocolitis [32]. Moreover, in addition to discordance between the symptoms and the degree of GI inflammation, endoscopic findings do not correlate with remission. Histologic findings may be a better indicator of remission, and this should be studied further. The histopathologic spectrum of ICI-induced colitis overlaps with findings that have traditionally been associated with IBD [33]. This is in concordance with the pathologic findings reported in the current case series. The addition of probe-based confocal laser endomicroscopy, which is currently experimental in the setting of IBD, could potentially be of value when clinical and endoscopic findings seem discordant, allowing in vivo microscopic assessment of the mucosa [34], although this remains to be studied. However, it is noteworthy to mention that histologic remission is difficult to achieve when post-treatment pathologic findings reveal persistent inflammation, as was evident in the current case series. This suggests that endoscopic as well as symptomatic remission should be assessed, and thus endoscopic features should be identified that can be used to guide treatment decisions, particularly early initiation of add-on therapies such as infliximab or vedolizumab and re-initiation of ICI therapy.

Noteworthy, patients who failed a trial of infliximab treatment before vedolizumab were at higher risk for receiving long duration of steroids and having lower response to vedolizumab as well. Also, some patients have persistence of endoscopic or histologic features with resolution of clinical symptoms. This observation stands true in patients with IBD, where it takes longer duration for histologic and endoscopic features to resolve compared with clinical symptoms. Additionally, a possible reason behind these findings could be the presence of factors that play a role in the pathogenesis of ICI-related colitis, such as fecal microbiome as proposed by other studies [35, 36]. The speculation that microscopic colitis is more refractory to treatment was not confirmed by this case series, where found similar success rate of both groups.

In addition to the above mentioned parameters, fecal levels of calprotectin, a calcium-binding protein that is derived from white blood cells, have been shown to correlate with GI inflammation, with favorable sensitivity and specificity [37–39]. Adding fecal calprotectin to the routine tests that are done when a GI-irAE is suspected would enable use of fecal calprotectin as a simple yet potentially valuable marker of response to treatment for colitis. The finding of lower mean fecal calprotectin levels in our patients who successfully achieved clinical remission compared with those who did not highlights the potential of fecal calprotectin in aiding management decisions, a claim that needs to be validated in further large-scale studies. In our cohort, we observed a steeper decline of calprotectin values in patients who received vedolizumab or infliximab within 14 days of onset compared with patients who received it after 14 days. Therefore, the early introduction of vedolizumab treatment before failure of other agents can be the optimal approach to decrease exposure to systemic immunosuppression. In addition, the finding of higher effectiveness in patients who received vedolizumab without failing a previous trial of infliximab supports this recommendation. The efficacy of concurrent vedolizumab and infliximab use has not been tested yet; this potential approach could prove to be efficacious, however the risk of unfavorable adverse effects is expected to be higher with combination of two potent immunosuppressants compared with only one of them. Short duration of induction steroid treatment however could still be needed to facilitate a speedy resolution of symptoms, especially that vedolizumab might need financial approval for this indication. Further prospective studies are needed to assess the efficacy of vedolizumab as a first line agent without steroid therapy.

## Conclusions

In conclusion, we believe that vedolizumab is appropriate for the treatment of steroid-refractory ICI-induced colitis, with favorable outcomes and a good safety profile. The current study is limited by its retrospective nature. In addition, this was a small series with a limited sample size, which did not allow us to deduce any strong claims. Future well-designed prospective studies are needed to compare current treatment strategies with vedolizumab to further delineate the utility of this treatment strategy, with a focus on optimal dosing practices while appropriately establishing the adverse event profile in larger cohorts. Comparative studies of vedolizumab and infliximab are needed to evaluate the efficacy and safety of these agents in ICI-induced colitis. Additionally, irAE treatment strategies, both present and emerging, should be assessed in the context of the impact of these strategies on histologic remission, overall survival, and the ability to restart ICI therapy.

## Additional file

Additional file 1: **Table S1.** Common Terminology Criteria of Adverse Events v5.0 grading for diarrhea and colitis. Table 2 Characteristics of patients grouped by infliximab and vedolizumab. Table S3 Characteristics by colitis stratified by the histology. (DOCX 21 kb)

## Abbreviations

CRP: C-reactive protein
CTCAE: Common Terminology Criteria for Adverse Events
CTLA-4: Cytotoxic T-lymphocyte activator-4
GI: Gastrointestinal
IBD: Inflammatory bowel disease
ICI: Immune checkpoint inhibitor
IMDC: Immune-mediated diarrhea and colitis
IQR: Interquartile range
irAE: Immune-related adverse event
PD-1: Programmed cell death protein 1
PD-L1: Programmed cell death ligand 1
SD: Standard deviation
