# Supplementary material for: Outcomes of vedolizumab therapy in patients with immune checkpoint inhibitor–induced colitis: a multi-center study
Source: J Immunother Cancer. 2018 Dec 5;6:142. doi: 10.1186/s40425-018-0461-4 (PMC6280383; doi:10.1186/s40425-018-0461-4)
Supplement: Supplementary file 1 — Table S1. Common Terminology Criteria of Adverse Events v5.0 grading for diarrhea and colitis. Table 2 Characteristics of patients grouped by infliximab and vedolizumab. Table S3 Characteristics by colitis stratified by the histology. (DOCX 21 kb) [file 40425_2018_461_MOESM1_ESM.docx]

**Additional file 1: Table S1** Common Terminology Criteria of Adverse Events v5.0 grading for diarrhea and colitis.

| **Gastrointestinal disorders** | | | | | |
| --- | --- | --- | --- | --- | --- |
|  | **Grade** | | | | |
| **Adverse Events** | **1** | **2** | **3** | **4** | **5** |
| Diarrhea | Increase of <4 bowel movements per day over baseline; mild increase in ostomy output compared to baseline | Increase of 4 - 6 bowel movements per day over baseline; moderate increase in ostomy output compared to baseline | Increase of ≥7 bowel movements per day over baseline; incontinence; hospitalization indicated; severe increase in ostomy output compared to baseline; limiting self-care ADL | Life-threatening  consequences; urgent intervention indicated | Death |
| Colitis | Asymptomatic; clinical or diagnostic observations only;  intervention not indicated | Abdominal pain; mucus or blood in stool | Severe abdominal pain; change in bowel habits;  medical intervention indicated;  peritoneal signs | Life-threatening  consequences; urgent intervention indicated | Death |

Table S2 Characteristics of patients grouped by infliximab and vedolizumab.

| Characteristic | Infliximab + Vedolizumab,  No. (%) | Vedolizumab only, No. (%) |
| --- | --- | --- |
| Total no. of patients | 9 | 19 |
| Checkpoint inhibitor type |  |  |
| CTLA-4 | 3 (33) | 5 (26) |
| PD-1/L1 | 2 (22) | 10 (53) |
| Combination | 4 (44) | 4 (21) |
| Mean duration of steroid therapy, days (SD) | 131 (74) | 85 (75) |
| Median time from symptom onset to vedolizumab/infliximab therapy, days (IQR) | 39 (6-152) | 15 (4-74) |
| Median no. of vedolizumab doses (IQR) | 3 (1-4) | 2 (1-4) |
| Mean fecal calprotectin level at time of onset µg/g (SD) | 268 (244) | 346 (290) |
| Peak grade of diarrhea |  |  |
| 2 | 5 (55) | 10 (53) |
| 3-4 | 4 (44) | 9 (47) |
| Initial endoscopic findings |  |  |
| Ulceration | 2 (22) | 6 (32) |
| Nonulcerative inflammation | 4 (44) | 9 (47) |
| Normal | 3 (33) | 4 (21) |
| Initial histologic findings |  |  |
| Active features | 5 (55) | 11 (57) |
| Chronic features | 1 (11) | 1 (5) |
| Microscopic | 3 (33) | 7 (37) |
| Mean overall duration of disease months (SD) | 7 (4) | 5 (3) |
| Mean fecal calprotectin level after vedolizumab therapy µg/g (SD) | 253 (344) | 165 (133) |
| Clinical remission | 6 (67) | 18 (95) |
| Last repeat endoscopic findings^a^ |  |  |
| Ulceration | 0 (0) | 1 (5) |
| Nonulcerative inflammation | 2 (22) | 5 (26) |
| Normal | 6 (67) | 3 (16) |
| Endoscopic remission | 4 (44) | 5 (26) |
| Active features on last repeat histologic analysis^a^ | 6 (67) | 6 (32) |

Abbreviations: SD, standard deviation; IQR, interquartile range.

^a^Repeat endoscopic and histologic evaluations were performed in 17 patients.

Table S3 Characteristics by colitis stratified by the histology.

| Characteristic | Microscopic colitis,  No. (%) | Active and chronic colitis, No. (%) |
| --- | --- | --- |
| Total no. of patients | 10 | 18 |
| Mean duration from ICI to onset (SD) | 220 (185) | 77 (88) |
| Checkpoint inhibitor type |  |  |
| CTLA-4 | 1 (10) | 7 (39) |
| PD-1/L1 | 8 (80) | 4 (22) |
| Combination | 1 (10) | 7 (39) |
| Mean duration of steroid therapy, days(SD) | 83 (67) | 113 (83) |
| Infliximab | 3 (30) | 6 (33) |
| Median time from symptom onset to vedolizumab/infliximab therapy, days (IQR) | 35 (7-152) | 12 (4-51) |
| Median no. of vedolizumab doses (IQR) | 3 (1-4) | 3 (1-4) |
| Mean fecal calprotectin level at time of onset µg/g (SD) | 121 (38) | 426 (287) |
| Peak grade of diarrhea |  |  |
| 2 | 5 (50) | 10 (56) |
| 3-4 | 5 (50) | 8 (44) |
| Initial endoscopic findings |  |  |
| Ulceration | 0 (0) | 8 (44) |
| Nonulcerative inflammation | 4 (40) | 9 (50) |
| Normal | 6 (60) | 1 (6) |
| Mean overall duration of disease months (SD) | 8 (2) | 6 (5) |
| Mean fecal calprotectin level after vedolizumab therapy µg/g (SD) | 154 (121) | 230 (295) |
| Clinical remission | 9 (90) | 15 (83) |
| Last repeat endoscopic findings^a^ |  |  |
| Ulceration | 0 (0) | 1 (6) |
| Nonulcerative inflammation | 4 (40) | 3 (17) |
| Normal | 2 (20) | 7 (39) |
| Active features on last repeat histologic analysis^a^ | 4 (40) | 8 (44) |

Abbreviations: SD, standard deviation; ICI, immune checkpoint inhibitor; IQR, interquartile range.

^a^Repeat endoscopic and histologic evaluations were performed in 17 patients.
